# Carotid plaque fissure: An underestimated source of intraplaque hemorrhage

**DOI:** 10.1016/j.atherosclerosis.2016.09.069

**Published:** 2016-09-28

**Authors:** Mat J. Daemen, Marina S. Ferguson, Frank J. Gijsen, Daniel S. Hippe, M. Eline Kooi, Kevin Demarco, Allard C. van der Wal, Chun Yuan, Thomas S. Hatsukami

**Affiliations:** a Department of Pathology, Academic Medical Center, Meibergdreef 9, 1105AZ, Amsterdam, The Netherlands; b Department of Radiology, University of Washington, 1959 NE Pacific Street, SS-202, Seattle, WA 98195-7117, USA; c Biochemical Engineering, Thoraxcenter, Erasmus Medical Center, Rotterdam, PO Box 2040, 3000 CA, Rotterdam, The Netherlands; d Department of Radiology, Maastricht University Medical Center, PO Box 5800, 6202 AZ, Maastricht, The Netherlands; e Walter Reed National Military Medical Center, 8901 Wisconsin Avenue, Department of Radiology, Building 9, Room 1799, Bethesda, MD 20889, USA; f Department of Surgery, Division of Vascular Surgery, University of Washington, Vascular Imaging Laboratory, 850 Republican Street, Brotman Building Room 124, Seattle, WA 98019, USA

**Keywords:** Hemorrhage, Plaque, Atherosclerosis

## Abstract

**Background and aims:**

Plaque fissuring, a phenomenon morphologically distinct from the classical rupture of a thinned fibrous cap, has not been well characterized in carotid atherosclerosis. The aim of this study was to establish the prevalence of plaque fissures in advanced carotid plaques with an otherwise intact luminal surface, and to determine whether they might be a source of intraplaque hemorrhage (IPH).

**Methods:**

We evaluated 244 surgically intact, ‘en bloc’ embedded, serially sectioned carotid endarterectomy specimens and included only those plaques with a grossly intact luminal surface.

**Results:**

Among the 67 plaques with grossly intact luminal surface, cap fissure was present in 39 (58%) plaques. A total of 60 individual fissures were present, and longitudinally mean fissure length was 1.3 mm. Most fissures were found distal to the bifurcation (63%), proximal to the stenosis (88%), and in the posterior (opposite the flow divider) or lateral quadrants (80%). 36% of the fissures remained in the superficial third of the plaque. 52% extended from the lumen surface to the middle third of the plaque and 12% reached the outer third of the plaque on cross section. Fissures often occurred between two tissue planes and were connected to IPH (fresh: 63%; any type: 92%) and calcifications (43%). No correlation was found with patient characteristics such as symptom status, carotid stenosis, hypertension, diabetes, smoking and medications (statins or antiplatelet agents).

**Conclusions:**

Plaque fissures are common in advanced carotid plaques with an otherwise grossly intact luminal surface and are associated with fresh intraplaque hemorrhage. As they occur on the interface between plaque components with different mechanical properties, further biomechanical studies are needed to unravel the underlying failure mechanisms.

## 1. Introduction

Fibrous cap rupture is one form of luminal surface disruption and is defined as a structural defect (a gap) in the fibrous cap that separates the lipid-rich necrotic core of a plaque from the lumen of the artery [1]. However, Michael Davies wrote in 1996 [2] that “There is a broad spectrum of both the severity and the morphology of plaque disruption episodes. At the minor end of the scale, the break in the cap is small, and although the plaque is expanded by blood within the core, the overall shape of the plaque is retained. More significant tears allow sufficient blood to enter the core so that stenosis rapidly increases.” The minor luminal surface disruptions that otherwise do not alter the overall luminal shape have also been referred to as plaque fissures. While fissures have been described in coronary atherosclerosis [3,4], its occurrence in carotid atherosclerosis is not well characterized. We hypothesized that plaque fissures do also occur in carotid atherosclerosis and are a possible source of carotid IPH. Prior studies have shown that IPH is associated with high-risk atherosclerotic carotid plaque, a faster plaque progression and the development of future ischemic cerebrovascular events [5–8]. To test our hypothesis, we quantified the presence of plaque fissures in carotid artery plaques with an otherwise intact luminal surface and examined the relation of these fissures with IPH and other plaque features in a consecutive series of 244 surgically intact, ‘en bloc’ embedded, and serially sectioned carotid endarterectomy specimens.

## 2. Materials and methods

### 2.1. Patient selection

Patients scheduled for carotid endarterectomy at either the University of Washington Medical Center or VA Puget Sound Health Care System, Seattle, Washington were recruited for this study. Institutional review boards at each facility approved the consent forms and study protocols. Subjects either had a transient ischemic attack or stroke ipsilateral to the index carotid artery within six months of their surgery and had ≥50% carotid stenosis or were asymptomatic with ≥80% carotid stenosis.

### 2.2. Tissue collection and processing

After informed consent, plaques were surgically removed en bloc by incising the carotid artery longitudinally along the vessel through the adventitia and outer media, but not into the plaque. The endarterectomy plane was developed around the plaque, which was divided proximally and distally with minimal surgical manipulation. In this way, the plaque wall and lumen were never violated. Typically, less than one millimeter (mm) of the outer wall of the artery was left behind. The specimens were gently flushed with Ringers lactate and placed in 10% neutral buffered formalin for a minimum of 24 h. They were then decalcified in 10% formic acid, processed and embedded en bloc in paraffin. The blocks containing the intact carotid plaque specimen were serially sectioned at a thickness of 10 μm at 1 mm intervals in the common carotid artery (CCA) and 0.5 mm intervals from the bulb through the internal carotid artery (ICA). The sections were stained with hematoxylin and eosin (H&E) or Mallory’s Trichrome.

### 2.3. Inclusion criteria

The first screening of the luminal shape of the plaques was of a low power (10×) computerized photograph of the H&E of each slice taken at either 1 mm (in the common carotid) or 0.5 mm (in the internal carotid) throughout the plaque. According to the definition of Michael Davies [2], only plaques with a retained overall shape of the lumen were included in the analysis. In these plaques, fissures were identified and plaque features adjacent to or near fissures were recorded. Plaques with other surface disruptions, such as large cap ruptures, ulcerations, protruding luminal thrombus, protruding calcifications, calcium nodules, and surgical disruptions, were excluded from further analysis.

### 2.4. Classification of histological features

Plaques with a retained luminal shape were microscopically scored for the presence or absence of fissures. Cap fissures were defined as a non-artefactual discontinuity of the luminal tissue planes containing red blood cells and macrophages between the layers. Fissure and plaque characteristics were scored by consensus by two experienced vascular pathologists (MF and MD) for the following: i) location of the fissure: CCA or ICA, upstream or downstream of the stenosis, location of the fissure as per quadrant (with the anterior quadrant containing the flow divider, the posterior quadrant across from the flow divider and the lateral and medial quadrants oriented as to the side of the carotid.); ii) relation with the shoulder of the lesion; iii) longitudinal track of the fissure, cross sectional track of the fissure, defined by the greatest depth of the fissure into the intima (superficial third, middle third, or outer third); iv) longitudinal length of the fissure, defined as the distance between the first and last cross-sections where the fissure was visible; v) relation with plaque composition i.e. luminal calcifications, loose extracellular matrix (=repair), microvessels, inflammation, intraplaque haemorrhage acute (fresh), intraplaque haemorrhage subacute and chronic (old), and mural thrombus. Each fissure was scored separately as one plaque might contain multiple fissures.

### 2.5. Statistical analysis

Categorical features of fissures were summarized as count (percentage). Clinical characteristics were compared between patients with and without fissures presence using Fisher’s exact test or the Mann-Whitney test. Patients missing a value for a clinical characteristic were excluded from analyses involving that characteristic but were retained for other analyses. All statistical calculations were conducted with the statistical computing language R (version 3.1.1; R Foundation for Statistical Computing, Vienna, Austria). Throughout, two-tailed tests were used with statistical significance defined as *p* < 0.05.

## 3. Results

Two hundred and ninety-four consecutive carotid endarterectomy specimens collected between 1998 and 2013 were investigated. Twenty-four specimens were excluded because of surgical disruption, another 26 were excluded because they were not processed to histology sections. Of the remaining 244 evaluable specimens, 100 had disrupted luminal surfaces because of one or more ulcerations, 25 had fibrous cap ruptures, 39 had protruding calcium nodules and 13 had mural thrombi (some specimens had multiple surface defects). The remaining 67 specimens with grossly intact and undistorted lumens were microscopically examined for the presence of fissures.

**Fig. 1:**
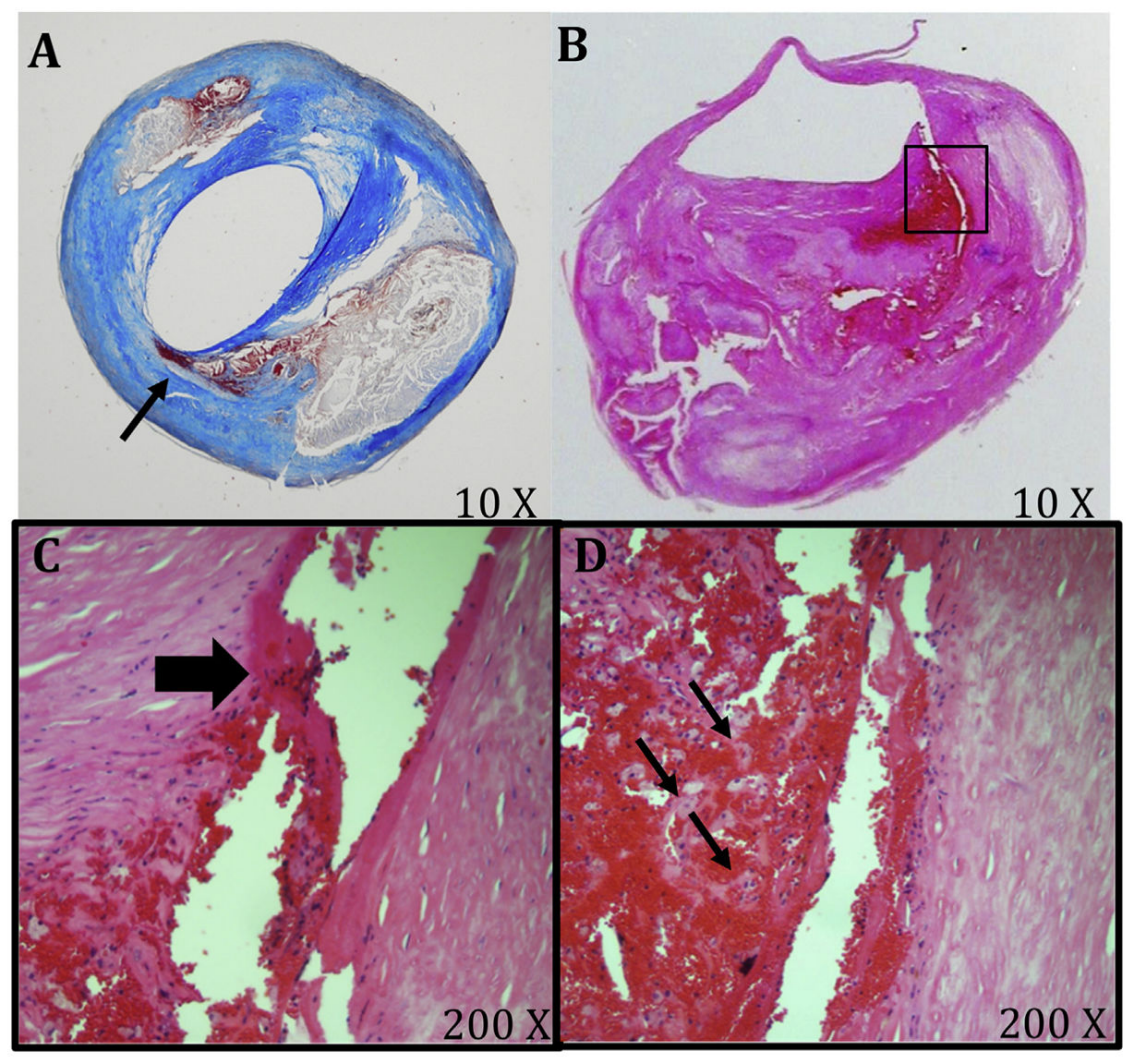
Fissures in carotid plaques (A) Mallory’s Trichrome stained carotid plaque with an intact luminal surface and a cap fissure containing intraplaque hemorrhage (arrow). (B) H&E stained internal carotid artery plaque with fissure at shoulder (different plaque than panel A). A luminal tear lifts a layer of the intima from the underlying fibrous tissue. (C and D) Magnification of region in panel B with the hemorrhage tract (block arrow) which extends into the necrotic core, and macrophage/foam cells (small arrows).

**Fig. 2:**
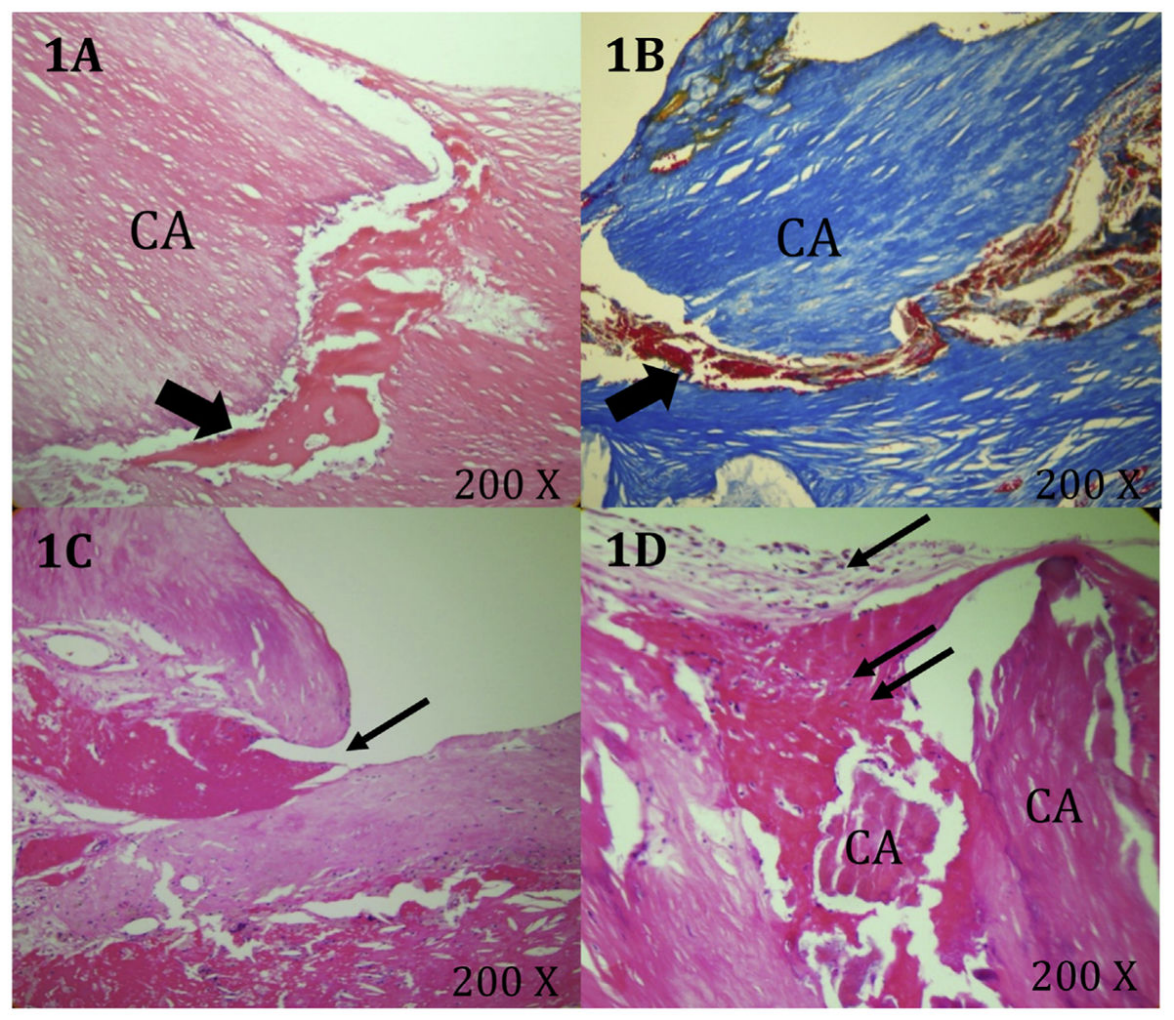
Characteristics of the tissues surrounding plaque fissures (A and B) H&E and Mallory’s stained plaque showing hemorrhage (arrows) dissecting the tissue planes between matrix and calcifications. (C) H&E stained plaque with fissure containing a small luminal fibrin thrombus (arrow). (D) Loose matrix (arrow) and thrombus (double arrow) covering the fissure at the luminal surface.

Of the 67 plaques analysed, 39 (58%) had at least one fissure (60 total) and 28 plaques had no fissures. Examples of plaque fissures are shown in Figs. 1 and 2. Twenty-four plaques had only one fissure, 11 plaques had two fissures, three plaques had three fissures and one plaque had five fissures (Table 1). Of the 60 individual fissures, the length along the lumen extended up to 5.5 mm (mean: 1.3 mm). Most of the fissures (63%) were found distal to the bifurcation, 88% were present proximal to the point of maximal stenosis, 80% were located in the posterior (opposite the flow divider) or lateral quadrants, and 45% were found in the shoulder regions of the plaque. Fissures in the shoulder regions were characterized by the presence of a luminal tear lifting a layer of the intima from the underlying fibrous tissue (Fig. 1B) and a hemorrhage tract extending into the necrotic core (Fig. 1C), a tract often lined by macrophages (60%) (Fig. 1D) or showed a hemorrhage dissecting the tissue planes and extending into the matrix around calcifications (Fig. 2A and B).

To determine the depth of fissures, cross sections of plaques were divided into thirds, with surface, middle, and outer layers. Twenty-two (37%) of the fissures remained within the superficial third of the plaque. Thirty-one (52%) extended from the lumen surface to the middle third of the plaque and seven (12%) reached the outer third of the plaque on cross section (Table 1).

Sixty-three percent of the 60 individual fissures were connected to fresh IPH and 92% were connected to IPH of any stage (Table 1). In contrast, only 25% of fissures were connected to areas of microvasculature. Of 39 plaques with at least one fissure, all contained IPH (fresh or old), compared with 25 of 28 (89%) plaques without an identified fissure (*p* = 0.07 for the difference). However, fresh IPH specifically was predominantly found in the fissured plaques (34 of 39, 88%) rather than non-fissured plaques (11 of 28, 39%) (*p* < 0.001 for the difference).

Forty-three percent of fissures were associated with regions of plaque calcification. Fissures connected to calcified aggregates typically did not originate from the shoulder regions and did not show a luminal tear lifting a layer of intima from the underlying fibrous tissue. Those fissures showed hemorrhage dissecting the tissue planes and extending into the matrix around the calcifications (Fig. 2A and B).

Small luminal thrombi (Fig. 2C) were present in only seven fissures (12%), while luminal loose matrix covering the ostium of the fissures (Fig. 2D) was present in 13 (22%), indicating that cap fissuring is a dynamic process.

Amongst the 28 plaques that did not contain a fissure, 18 (64%) were complex lesions with necrotic core and calcification. Ten (36%) were simple fibrolipid plaques, with the remaining four containing large areas of calcification.

### 3.1. Clinical characteristics

Clinical characteristics of the 67 patients are summarized in Table 2. Data collection was largely complete in 64 patients and partial in three patients. Of the 67 patients, 91% (61) were men and the mean age was 67 years. Seventy percent of the patients were asymptomatic and 80% had a carotid stenosis higher than 80%. History of hypertension was prevalent (86%) and 65% were on statins. There were no statistically significant differences in the collected clinical characteristics between patients with and without plaque fissures.

## 4. Discussion

Plaque fissures were very common in this group of advanced carotid artery plaques with grossly intact and undistorted lumens, occurring in 58% of such specimens. As we did not include the plaques with a distorted luminal shape secondary to luminal thrombus, fibrous cap rupture, ulceration or protruding calcified nodules, the prevalence of fissures in advanced carotid plaque may be even higher.

Cap fissuring appears not to be a random process as many fissures occur in the shoulder regions of the plaque and the majority of the fissures were found proximal to the stenosis (88%). Asymmetry of proximal and distal plaque features has been described earlier. Dirksen et al. [9] reported that approximately two-thirds of the carotid plaques showed more macrophages and less smooth muscle cells upstream and proximal to the point of maximal stenosis. Cicha et al. [10] showed that 86% of carotid plaque ruptures were observed upstream from the stenosis and also noted increased longitudinal asymmetry of the thickness of the fibrous cap and lipid core in ruptured plaques.

This predilection of the proximal parts of the carotid plaques for plaque ruptures and fissures, as presented here, suggests a major role for biomechanical factors. The occurrence of cap fissures along the interface between plaque components with different mechanical properties, e.g. between fibrous tissue and a soft necrotic core or between fibrous tissue and a stiff calcified inclusion further supports a biomechanical cause of plaque fissure.

**Fig. 3:**
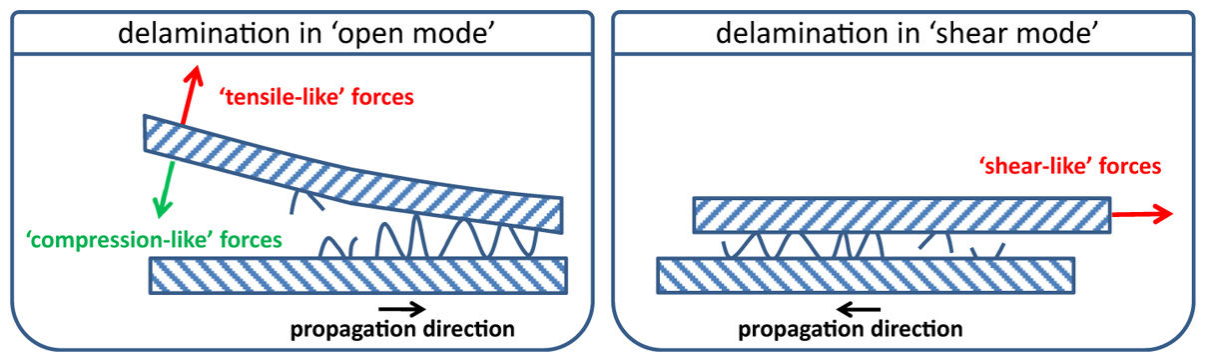
Schematic drawing of the two modes of delamination Delamination in the open mode (left panel) with tensile-like forces (blue arrow) and compression-like forces (red dashed arrow) and in the shearing mode (right panel).

The underlying biomechanical failure mechanism between layers with different properties is called delamination. Two delamination modes are generally discerned: delamination in the open mode and delamination in the shear mode [11]. In the open mode, also referred to as peeling, the layers are separated by a force directed normal to the interface between the plaque components (Fig. 3), and therefore normal to the fissure propagation direction. Peeling will only occur if a tensile-like force, tearing the layers apart, is exerted on the interface. If the force is directed in the other direction –a compression-like force – delamination in the open mode will not occur. In the shearing mode, the layers are separated by a shear-like force directed parallel to the interface, and therefore parallel to the fissure propagation direction. Although the 3D stress distribution in the plaque is highly complex, compressive-like forces can be expected to be dominant in the radial direction (which is roughly normal to the orientation of the interfaces), and peeling is not likely to occur. As fissures in the carotid plaques were associated with longitudinally running extracellular matrix fibers in the shoulder regions, and at the border of loose matrix and calcium deposits, shearing is probably the most dominant delamination mode in carotid plaques.

Delamination is also associated with other vascular pathologies. In aortic dissections, delamination between the wall layers was studied in the context of collagen microstructure [12] and it was shown that delamination might play an important role in plaque detachment at the shoulder regions during balloon angioplasty [13]. Furthermore, delamination is associated with the increased thoracic aneurysms in Marfan patients [14].

Like other failure mechanisms, cap fissure or delamination is initiated when a local mechanical stress exceeds a certain threshold value, called delamination strength. Initiation of the fissure does not necessarily occur exclusively at the luminal side. In a recent study, it was shown that the highest mechanical stress in coronary plaques is often located at the interface between the cap and the necrotic core [15], making this location another potential source of the fissure. Once initiated, fissures will easily propagate along the interface towards the luminal side, since mechanical stresses at the lumen-wall interface are generally expected to be relatively high and rapidly decrease in the deeper arterial structures [15,16], halting fissure propagation and explaining our observation that deeper layers contain fewer fissures.

The upstream prevalence of fissures matches the upstream prevalence of cap ulcerations [10,17,18]. The observations are most probably attributable to the increased macrophage density in upstream plaque regions [9,10]. Macrophages are associated with enzymatic activities leading to degradation of collagen and reduced delamination strength and cap strength [19]. The higher incidence of fissures in the posterior and lateral quadrant might be attributable to absence of supporting tissue surrounding the plaque. This potentially leads to increased plaque deformation in that quadrant and hence increased mechanical stress.

We have seen evidence of surface repair of fissures, as seven out of 60 (12%), showed a small luminal fibrin thrombus (Fig. 2C) and 13 out of 60 (22%) showed loose extracellular matrix covering the fissure at the luminal surface (Fig. 2D). This indicates that plaque fissuring is a dynamic process. The majority of the fissures, however, did not show features of repair. Although fissures are at the minor end of the scale of plaque rupture, it is possible that these small disruptions can develop into major ruptures due to luminal discontinuity and certainly due to the influx of blood into the plaque. This enforces the need to better understand the driving forces that fissure a plaque and those that make it heal.

Although we are the first to extensively report on the features of fissures in carotid artery plaques, plaque fissuring is not a recent discovery as it was described in the coronaries as early as the 1930s [20]. A 1966 paper by Constantinides describes an impressive serial section study of occluded arterial segments from 20 consecutives cases of coronary thrombosis in which every thrombus was anchored in cracks of the surrounding atheromatous wall and most of the accompanying plaque haemorrhages could be traced to an entry of blood through similar breaks in the atherosclerotic lining of the coronary vessel wall [21]. In 1985, Michael Davies also described coronary artery fissures in 21% of the patients that died of ischemic heart disease [3], while Falk and Virmani recently reported a 10–15% frequency of fissuring in coronary arteries [4].

We show here that fissures are also quite frequent in carotid artery endarterectomy specimens with an intact, undistorted lumen. The actual numbers may even be higher had we not used a stringent selection criterion such as a retained luminal shape. As described for the coronary artery, fissures in our study were often (63%) connected to fresh IPH and fresh IPH was significantly more common in fissured plaques than non-fissured plaques. Thus, besides highly permeable intraplaque vessels and the well-known rupture of a thin-cap fibroatheroma, fissures may well be another cause of IPH in carotid arteries.

We did not observe clinical associations, most probably due to the low number of cases of a selected study population, as we only included the subset of plaques with an intact luminal shape. Furthermore, many of the clinical risk factors, including hypertension, were highly prevalent. This fact highlights the need to develop high resolution imaging techniques to identify plaque fissures in individuals at an earlier stage of disease in the broader population.

Prospective longitudinal MRI studies of the carotid artery have shown that IPH is associated with a more rapid plaque progression that appears to be resistant to statin therapy [5,22], and poses an increased risk of future stroke or transient ischemic attack [8,23]. Our data show that 92% of the fissures are associated with haemorrhage, suggesting that fissures in carotid plaques need to be taken into account as a possible and yet unrecognized cause of IPH. Although one might intuitively expect that the fissures are closed in vivo, this is not necessarily the case. Due to intraluminal blood pressure, carotid plaques expand in the circumferential direction. Fissures can therefore be expected to be open, at least during part of the heart cycle. When the fissure is open, the gap of the fissure will be widest at the luminal side, decreasing in width when extending further into the plaque. Since the fissure is widest at the luminal side, a significant axial velocity component of the blood will be present. The typical speckle appearance of flowing blood in intravascular ultrasound in coronary plaque fissures supports this hypothesis [24,25]. This constant replenishment from the lumen explains our findings of fresh erythrocytes at the luminal end of the fissures. Deeper in the fissure, the opening gets smaller, and the blood is constrained by the surrounding tissue. In the absence of an endothelial cell layer, diffusion of erythrocytes into the plaque can be expected, along with the usual accompanying inflammatory response. Since velocities associated with diffusion of erythrocytes will be extremely low, quantification of this process through imaging is extremely challenging. These mechanistic arguments in favour of a diffusion of erythrocytes from the fissured lumen into the plaque and our observations that fissures can heal, and can also reopen, may explain the consistent presence of IPH in carotid plaque imaged over a period of four years [26]. As small fissures that are not associated with larger IPH cannot currently be visualized with non-invasive imaging techniques such as ultrasound, MRI and CT, techniques with higher resolution and sensitivity need to be developed. These tools will allow answering several important questions such as whether fissures represent an early stage in the development of larger IPH, if and how fissures contribute to IPH progression, and whether the identification of the early presence of fissures presents an opportunity to prevent progression to larger amounts of destabilizing intraplaque haemorrhage.

This study has a number of limitations. First, only advanced plaques removed through CEA were examined. Therefore, the fissures identified in this study may not be representative of fissures which may occur in plaques with an earlier stage of atheroma. In addition, only plaques with a smooth lumen–undistorted by fibrous cap rupture, ulceration, protruding thrombus or calcium, calcium nodule, or surgical disruption–were included as these features can confound identification of fissures. Although our surgical technique of incising only the adventitia and part of the media and not into the plaque itself leaves the lumen surface undisturbed, there is always the possibility that manipulation can separate tissue planes and introduce red blood cells. To mitigate the possibility of artefact confounding our assessment of fissures, we did not include potential fissures that contained only red blood cells but only included fissures that contained both red blood cells and macrophages. Lastly, this is a cross-sectional study which cannot establish any causal links between fissures and IPH or other features. We have hypothesized potential biomechanical mechanisms that relate plaque fissuring with IPH, but these remain to be proven in subsequent studies.

## Figures and Tables

**Table 1:** Characteristics of the fissures (N ¼ 60 fissures from 39 plaques).

Variable		No. (%)
Longitudinal length of fissure^a^	Single cross-section	15 (25.0%)
	0.5–1.0 mm	22 (36.7%)
	1.5–2.0 mm	14 (23.3%)
	2.5–5.5 mm	9 (15.0%)
**Location of fissures**		
Within the shoulder region		27 (45.0%)
Relative to bifurcation^b^	Proximal	26 (43.3%)
	Distal	38 (63.3%)
Relative to stenosis	Proximal	53 (88.3%)
	Distal	7 (11.7%)
Quadrant of cross-section^b^	Anterior (Flow divider)	8 (13.3%)
	Medial	14 (23.3%)
	Posterior	24 (40.0%)
	Lateral	28 (46.7%)
Greatest depth into the intima^c^	Surface layer	22 (36.7%)
	Middle layer	31 (51.7%)
	Outer layer	7 (11.7%)
**Plaque features associated with fissures**		
Any intraplaque hemorrhage		55 (91.7%)
Fresh intraplaque hemorrhage		38 (63.3%)
Lipid-rich necrotic core		37 (61.7%)
Macrophages		36 (60.0%)
Calcification		26 (43.3%)
Microvessels		15 (25.0%)
Loose matrix		13 (21.7%)
Thrombus		7 (11.7%)
Inflammatory infiltrate		3 (5.0%)

a Distance between the first and last cross-sections where the fissure was seen; sections are spaced every 0.5 mm or 1.0 mm.

b Categories not mutually exclusive because some fissures crossed the bifurcation and multiple quadrants.

c The cross-sectional area was divided into thirds to define the surface, middle, and outer layers.

**Table 2:** Clinical characteristics of the 67^a^ patients with carotid plaques with an intact surface.

Variable		Overall (N = 67^a^)	Presence of fissure		*p*-value	No. Available

			Yes (N = 39^a^)	No (N = 28^a^)		
Sex	Male	61 (91.0%)	35 (89.7%)	26 (92.9%)	>0.99	67
	Female	6 (9.0%)	4 (10.3%)	2 (7.1%)		
Age–years		67 (48–83)	68 (48–83)	66 (49–83)	0.74	66
Carotid clinical status	Symptomatic	19 (29.7%)	12 (31.6%)	7 (26.9%)	0.78	64
	Asymptomatic	45 (70.3%)	26 (68.4%)	19 (73.1%)		
Carotid stenosis	80–99%	51 (79.7%)	32 (84.2%)	19 (73.1%)	0.35	64
	50–79%	13 (20.3%)	6 (15.8%)	7 (26.9%)		
History of hypertension	Yes	55 (85.9%)	32 (84.2%)	23 (88.5%)	0.73	64
	No	9 (14.1%)	6 (15.8%)	3 (11.5%)		
History of diabetes mellitus	Yes	17 (26.6%)	11 (28.9%)	6 (23.1%)	0.77	64
	No	47 (73.4%)	27 (71.0%)	20 (76.9%)		
History of tobacco use	Current	24 (37.5%)	15 (39.5%)	9 (34.6%)	0.90	64
	Former/quit	25 (39.1%)	15 (39.5%)	10 (38.5%)		
	Never	15 (23.4%)	8 (21.1%)	7 (26.9%)		
Medications	Aspirin	45 (71.4%)	26 (70.3%)	19 (73.1%)	>0.99	63
	Clopidogrel or warfarin	14 (22.2%)	6 (16.2%)	8 (30.8%)	0.22	63
	Statins	42 (65.6%)	25 (65.8%)	17 (65.4%)	>0.99	64

Values are no. (%) or median (range) unless otherwise stated.

a Complete clinical data was not available for some patients. Specifically age was missing for one patient; symptom status, stenosis, hypertension, diabetes, tobacco use, and statins were missing for three patients; aspirin, clopidogrel, or warfarin was missing for four patients.
